# Genomic Epidemiology of Clinical *Klebsiella pneumoniae* in the Middle East and North Africa

**DOI:** 10.3390/antibiotics15040349

**Published:** 2026-03-29

**Authors:** Hamid Reza Sodagari, Rima D. Shrestha

**Affiliations:** 1 Department of Pathobiology, School of Veterinary Medicine, St. George’s University, St. George’s P.O. Box 7, Grenada; 2 Department of Internal Medicine, University of Illinois College of Medicine Peoria (UICOMP), Peoria, IL 61605, USA

**Keywords:** *Klebsiella pneumoniae*, Middle East and North Africa, MENA, SNP clusters, geographic distribution, ESBL

## Abstract

**Background**: *Klebsiella pneumoniae* is a Gram-negative bacterium that is found in human microbiota and in diverse environments. This opportunistic pathogen exhibits a highly variable genetic background and is responsible for a broad range of hospital- and community-acquired, multidrug-resistant infections worldwide. To track transmission pathways and understand genetic diversity, single-nucleotide polymorphism (SNP) clustering has become an essential tool. **Methods**: This study examines data from 2018 to 2024 in the NCBI Pathogen Detection database to determine the temporal and spatial distribution of SNP clusters in clinical *K. pneumoniae* across Middle East and North Africa (MENA) countries. **Results**: Among 1858 isolates, a heterogeneous population structure was observed. Of the 478 identified SNP clusters, a few dominant clusters accounted for 37% of the isolates, and numerous low-frequency lineages were detected. The descriptive yearly snapshot revealed a diverse representation of top clusters. Geographical analysis showed the presence of both localized and limited cross-border distribution patterns. Countries with diverse clusters also exhibit higher diversity of carbapenem- and ESBL-resistant genes. **Conclusions**: These findings provide valuable insights into the dominant, regionally concentrated *K. pneumoniae* lineage across MENA countries, assisting future genomic surveillance and efforts to combat clinical *K. pneumoniae* infections in this region.

## 1. Introduction

*Klebsiella pneumoniae* (*K. pneumoniae*) is a Gram-negative bacterium that is also commonly identified in the human flora and natural environments [1]. *K. pneumoniae* exhibits variability in its ability to colonize different sites of the body, including the skin and respiratory tract [2]. This opportunistic pathogen can thus cause several infections, including necrotizing pneumonia, lung abscesses, liver abscesses, endogenous endophthalmitis, bloodstream infections, and urinary tract infections [3,4]. Most *K. pneumoniae* infections are predominantly reported in the elderly, immunocompromised individuals, and newborns and are frequently associated with healthcare outbreaks worldwide [5,6,7], including the Middle East and North Africa (MENA) region [8,9,10,11]. Transmission occurs through contaminated medical devices, blood products [12,13], and direct contact with patients or healthcare personnel [14,15]. Intensive care unit (ICU)-associated infections, sepsis, and surgical site infections have also been widely documented [16,17,18]. Additionally, *K. pneumoniae* is also responsible for diseases in livestock [19,20,21,22] and birds [23], indicating zoonotic potential and a broad ecological niche. The clinical and ecological diversity of this pathogen underscored the need for genomic approaches to better understand its population structure and transmission dynamics.

The advancement of next-generation sequencing, such as whole-genome sequencing, and computational methods has significantly transformed our understanding of bacterial population structure and transmission dynamics. These technologies enable researchers to generate high-resolution data on virulence traits, antimicrobial resistance profiles, and genetic relationships across bacteria [24]. Comparative genomic analyses also elucidate genomic differences and the molecular mechanisms that drive phenotypic diversity and pathogenicity [25].

Genomic analysis of *K. pneumoniae* showed a flexible and diverse pangenome with several accessory genes [20]. This accessory genome plays a pivotal role in the emergence of high-risk antibiotic-resistant and hypervirulent isolates, increased pathogenicity, invasive infections, and rapid adaptation to specific niches or hosts [23,26]. For example, a *K. pneumoniae* genome investigation has revealed both rapid regional and international spread of specific *K. pneumoniae* strains [27] and highlights genomic epidemiology significance. Another study identified two major high-risk clonal groups among *K. pneumoniae* populations: (a) a hyper-virulent clonal complex responsible for community-acquired invasive infections, and (b) a multidrug-resistant clonal complex causing healthcare-related infections [28,29]. However, comparable genomic data from the MENA region remains limited.

Single-nucleotide polymorphism (SNP) refers to a specific position in a DNA sequence where different individuals exhibit variation at a single nucleotide [30]. SNPs represent the most common type of genetic variation among closely related microbial species, strains, or isolates [30,31]. SNP-based phylogenetics and pairwise distance matrices have become essential tools for characterizing bacterial population structures [32]. Population structure in bacterial genomics is largely inferred from SNP variation, enabling classification of genetically related isolates into distinct lineages. In hospital investigations of *K. pneumoniae*, SNP clustering has provided insights into the genetic background [33,34] and global dissemination of high-risk clones such as ST258/512, ST101, ST11, and ST307 [27].

The emergence of hypervirulent lineages (hvKp) associated with community-acquired invasive disease, such as pyogenic liver abscess, has limited treatment options for *K. pneumoniae* infections and increased global health risks [35]. Multidrug-resistant (MDR) *K. pneumoniae* infections, such as those resistant to extended-spectrum β-lactamase (ESBL), carbapenems, and colistin [36,37], also contribute to increased morbidity, mortality, and healthcare burden [38]. Reports of carbapenem-resistant *K. pneumoniae* in the MENA region highlight the growing prevalence of high-risk clones driven by core-genome mutations and horizontally acquired antimicrobial resistance (AMR) determinants [39,40,41,42]. An increasing prevalence of virulent and MDR strains, arising from both core-gene mutations and the buildup of horizontally acquired AMR determinants, has led the World Health Organization (WHO) to classify *K. pneumoniae* as a significant global health concern [25]. Identification of dominant SNP clusters, their geographical distributions, and associated resistant genes is thus essential for outbreak detection, epidemiological surveillance, and informing infection control strategies [43].

To identify population structure in clinical *K. pneumoniae* across the MENA region, we extracted and analyzed clinical *K. pneumoniae* data from 2018 to 2024 in the National Center for Biotechnology Information (NCBI) Pathogen Detection database (https://www.ncbi.nlm.nih.gov/pathogens/; accessed on 25 March 2026). This study aimed to (i) describe overall population structure among clinical *K. pneumoniae* isolates using SNP-based clustering across the MENA countries; (ii) identify dominant clustering and their prevalence; (iii) determine a descriptive yearly snapshot of cluster prevalence during the study period (2018–2024); (iv) investigate geographical distribution of the most prevalent clusters across the MENA countries; and (v) identify the prevalent carbapenem, ESBL, and colistin resistance genes per cluster. This study provides valuable insights into the dominant and regionally concentrated SNP clusters of the *K. pneumoniae* population in the MENA region, providing practical information for future genomic surveillance and efforts to combat *K. pneumoniae* infections in this region.

## 2. Results

### 2.1. Overall SNP Cluster Prevalence

Based on our analysis, 478 distinct SNP clusters were identified among clinical *K. pneumoniae* isolates collected from the MENA region between 2018 and 2024. Cluster sizes varied widely, ranging from 160 isolates (representing 8.6% of the isolates) to singleton clusters. The results revealed several dominant SNP clusters and numerous rare clusters (Table S2).

### 2.2. Top 10 SNP Clusters

**Figure 1:**
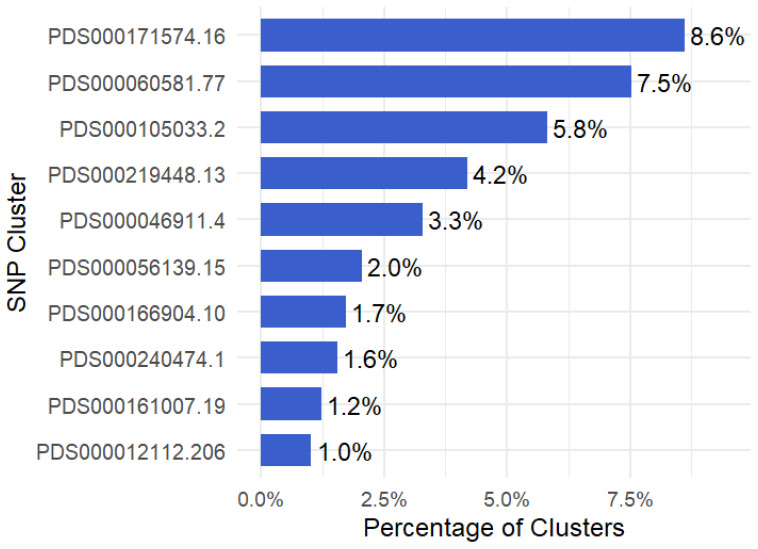
Prevalence of top 10 SNP clusters of *K. pneumoniae* in the MENA region (2018–2024). The y-axis represents SNP cluster IDs in the NCBI database, and the x-axis indicates the percentage of each cluster in the database.

Among the 478 identified SNP clusters, the ten most frequent ones accounted for 37% of all isolates. The largest cluster, PDS000171574.16, comprised 160 isolates (8.6% of the total), followed by PDS000060581.77 with 140 isolates (7.5%) and PDS000105033.2 with 108 isolates (5.8%) (Figure 1). The remaining top clusters ranged from 78 to 19 isolates, corresponding to proportions between 4.2% and 1.0% of the total isolates (Figure 1). These results indicate that a small subset of clusters dominates the population, while most clusters are comparatively rare.

### 2.3. Descriptive Yearly Distribution of Top 10 SNP Clusters

**Figure 2:**
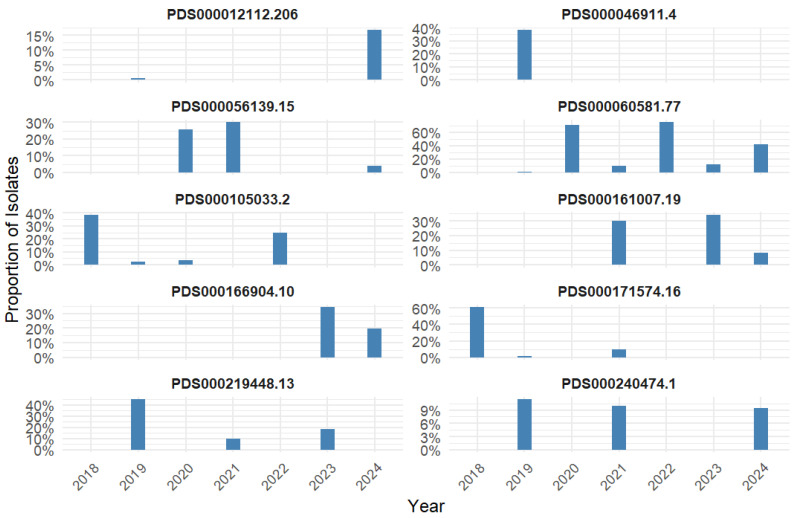
Prevalence of the top 10 SNP clusters among clinical *K. pneumoniae* isolates from MENA countries (2018–2024). The y-axis indicates the proportion of isolates per SNP cluster detected per year (x-axis).

The prevalence of the top 10 SNP clusters was calculated for each year of the study period (2018–2024) to provide a descriptive snapshot of temporal distribution (Figure 2, Table S3).

In 2018, two clusters, including PDS000171574.16 and PDS000105033.2, dominated, accounting for 61.3% and 38.7% of isolates, respectively. In 2019, three SNP clusters, including PDS000219448.13 (44.9%), PDS000046911.4 (38.6%), and PDS000240474.1 (11.4%), became the most prevalent, while several clusters, including PDS000012112.206 and PDS000060581.77, were detected at very low frequencies (<1%).

For 2020, PDS000060581.77 was the dominant cluster (71.1%), followed by PDS000056139.15 (25.6%). In 2021, the prevalence of clusters was spread across several low-frequency groups, with PDS000056139.15 and PDS000161007.19 each representing 30% of isolates, and PDS000060581.77 accounting for 10%.

Similarly, in 2022, PDS000060581.77 was the most prevalent cluster (75%), while PDS000105033.2 accounted for 25% of isolates. In 2023, prevalence was more balanced across PDS000161007.19 (34.4%), PDS000166904.10 (34.4%), PDS000219448.13 (18.8%), and PDS000060581.77 (12.5%). In 2024, similar to 2020 and 2022, PDS000060581.77 remained the most prevalent cluster (42.1%), followed by PDS000166904.10 (19.6%) and PDS000012112.206 (16.8%), with other clusters contributing smaller proportions.

Overall, the descriptive snapshots illustrate the fluctuating representation of the top 10 SNP clusters across the study period. Due to inconsistent SNP cluster detection for several years, these findings are presented as cross-sectional proportions, and no temporal trends were inferred.

### 2.4. Geographic Distribution of Top 10 SNP Clusters

**Figure 3:**
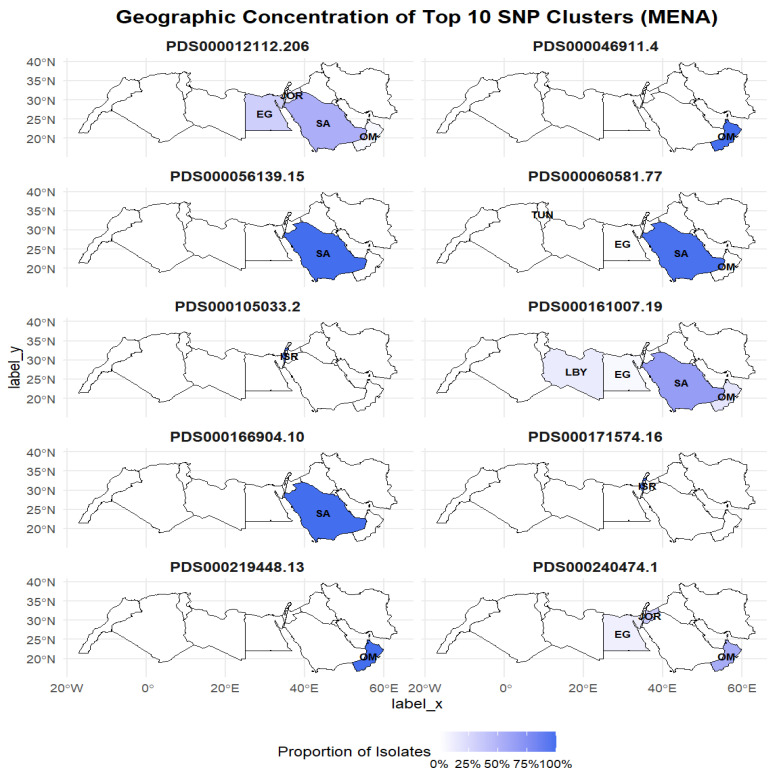
Geographical distribution of the top 10 SNP clusters of clinical *K. pneumoniae* isolated from MENA countries between 2018 and 2024. The MENA countries are described as SA: Saudi Arabia; OM: Oman; EG: Egypt; ISR: Israel; JOR: Jordan; LBY: Libya; TUN: Tunisia. Label_y in the y-axis is the latitude, and label_x in the x-axis is the longitude of the MENA.

We investigated the geographic distribution of the top 10 SNP clusters across MENA countries (Table 1, Figure 3).

Several clusters were highly localized to specific countries, which accounted for 100% of isolates in those clusters. PDS000046911.4 and PDS000219448.13 were identified only in Oman, whereas PDS000105033.2 and PDS000171574.16 were reported only in Israel. Two other clusters, including PDS000056139.15 and PDS000166904.10, were only found in Saudi Arabia.

Other clusters displayed a more dispersed distribution. PDS000012112.206 was detected in four countries of the MENA region, with the majority of isolates in Saudi Arabia (52.6%) and Egypt (31.6%), followed by Jordan (10.5%) and Oman (5.3%). Moreover, PDS000060581.77 was predominantly found in Saudi Arabia (97.9%), with very small proportions in Egypt, Oman, and Tunisia (each 0.7%). PDS000161007.19 was most prevalent in Saudi Arabia (65.2%), followed by Oman (17.4%), Libya (13.0%), and Egypt (4.3%). PDS000240474.1 was mainly detected in Oman (55.2%) and Jordan (34.5%), with a smaller proportion in Egypt (10.3%).

### 2.5. AMR Gene Distribution Across Top 10 SNP Clusters

**Figure 4:**
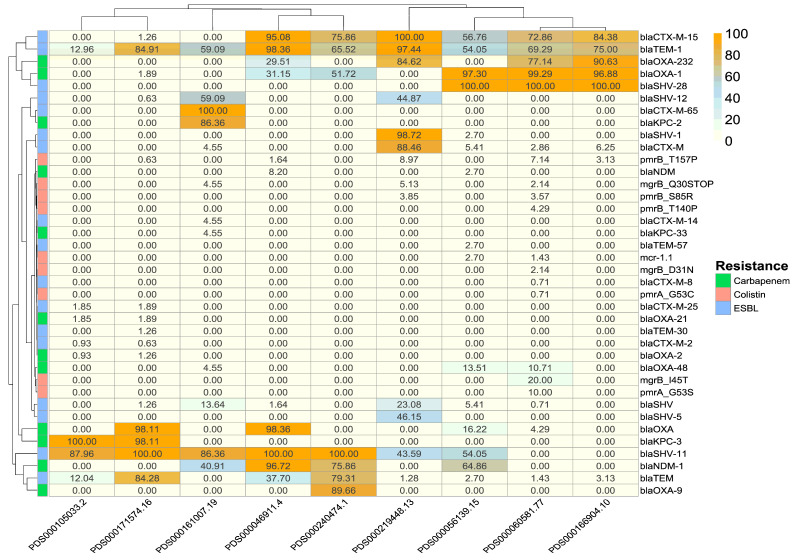
Heatmap showing the key antimicrobial resistance genes associated with extended-spectrum β-lactamase (ESBL), carbapenemase, and colistin resistance across the top 10 *K. pneumoniae* SNP clusters from the MENA region. The heatmap displays normalized counts (expressed as %) carrying each gene, with hierarchical clustering dendrograms for both SNP clusters (x-axis) and resistance genes (y-axis) to illustrate similarity in resistance profiles across clusters.

The heatmap shown in Figure 4 indicates distinct antimicrobial resistance gene carriage and cluster-relatedness in the top 10 *K. pneumoniae* SNP clusters. Highly abundant multiple resistance genes (*blaSHV-1*, *blaCTX-M-15*, and *blaNDM-1*) are predominantly observed in two SNP clusters, PDS000060581.77 and PDS000171574.16. The plasmid-mediated colistin resistance gene *mcr-1.1* was present in clusters PDS000060581.77 and PDS000056139.15. Chromosomal mutations associated with colistin resistance (*mgrB*- *Q30STOP*, *D31N*, *I45T*, *pmrA* and *pmrB*- *pmrB_T157P*, *pmrB_S85R*, *pmrB_T140P*) were predominantly observed in clusters with a high prevalence of carbapenemase and ESBL genes.

### 2.6. Multi-Locus Sequence Typing (MLST) Across Top 10 SNP Clusters

Our MLST analysis revealed that high-risk clones were detected in the top 10 SNP clusters, including PDS000171574.16 (ST258), PDS000161007.19 (ST11), PDS000046911.4 (ST11), and PDS000105033.2 (ST512). Other important clones, such as ST2096, were also identified in two of the top 10 clusters (PDS000060581.77 and PDS000166904.10). Table 1 shows the identified sequence type related to each of the top 10 SNP clusters.

## 3. Discussion

In the present study, we conducted genomic epidemiology analysis on *K. pneumoniae* isolates collected from the MENA region between 2018 and 2024. The prevalence, yearly distribution, and geographical distribution of SNP clusters in the pathogen were investigated in this study. Our findings offer critical insight into population structure, dominant clones, and the regional distribution of *K. pneumoniae*. The highly heterogeneous nature of the pathogen and the presence of country-specific clusters were among the most important findings of this study. The results further underscore the important role of genomic epidemiology as a complement to traditional microbiology. Genomic investigations provide the resolution required for detecting clonal spread, identifying outbreak strains, and tracking transmission chains that are not detectable by phenotype-based surveillance alone [44]. Implementing routine genomic studies in regional surveillance systems could enhance early detection of outbreaks and inform targeted infection control measures.

Based on our results, 478 distinct SNP clusters were identified, which indicates a highly heterogeneous population structure among *K. pneumoniae* isolates from the MENA region. The size distribution of clusters was heavily skewed. A small number of clusters (top 10) were prevalent, accounting for 37% of the isolates, while there were numerous clusters containing very few or even singleton isolates. This result is consistent with previous reports, showing the pathogen’s remarkable genomic plasticity and ability to adapt to diverse ecological environments [20]. Such heterogeneity may be driven not only by within-hospital evolution [45] but also by horizontal gene transfer events [46] and by the contribution of non-clinical reservoirs, such as the environment and food animal production chains, as highlighted by a recent study [47]. Including environmental and animal sampling in future surveillance would be beneficial to clarify the role of these reservoirs in shaping clinical diversity.

Our results showed the dominance of specific clusters, which suggests a small subset of the *K. pneumoniae* lineage was successful in colonizing the clinical setting in the MENA region. For instance, the top three clusters, including PDS000171574.16, PDS000060581.77, and PDS000105033.2, accounted for nearly 22% of isolates, showing their epidemiological significance. This finding aligns with the global pattern, which indicates that a limited number of high-risk clones often dominate clinical isolates [48]. Understanding why these clones succeed will require integrating genomic analyses with plasmid and virulence profiling, which has been shown to explain the expansion of globally successful lineages in a previous investigation [49]. Such complementary approaches may reveal whether dominance of specific SNP clusters is associated with antimicrobial resistance determinants, virulence factors, or specific clinical or environmental selection pressures.

We investigated the prevalence of each top SNP cluster in each year of the study period (2018–2024). Our findings showed that in 2018, two clusters, including PDS000105033.2 and PDS000171574.16, accounted for nearly all isolates that year, indicating early dominance of a specific lineage. In 2019, other SNP clusters such as PDS000046911.4 and PDS000219448.13 were dominant, which represented a temporal shift in the relative abundance of clones, consistent with previously reported temporal shifts in genomic subpopulations of *K. pneumoniae* [45]. These temporal fluctuations underscore the importance of longitudinal genomic surveillance, as cross-sectional snapshots may overlook transient expansions or transmission events. Continuous temporal trend investigations would enable more reliable trend analyses to identify dominant lineages over time and support timely recognition of outbreaks.

From 2020, PDS000060581.77 SNP cluster emerged as a consistently dominant cluster. This cluster accounted for 71% of isolates in 2020 and maintained high prevalence in the next years of the study period. This result coincides with the previous report of long-term persistence of a distinct *K. pneumoniae* subpopulation over nearly a decade [45]. It can be hypothesized that this cluster may have a successful lineage, which can be due to improved transmissibility, adaptability to diverse environmental conditions, or clinical fitness across healthcare settings in the MENA region. Further research is required to shed more light on the underlying reasons for the dominance and persistence of some clones over time. Overall, our yearly prevalence analysis highlighted the importance of conducting continuous cross-sectional and longitudinal investigations across different time periods to better understand the dynamics of clone emergence, persistence, and changes over time.

Our geographical distribution analysis revealed two main patterns among the top ten SNP clusters. Several clusters, including PDS000046911.4 (Oman), PDS000219448.13 (Oman), PDS000105033.2 (Israel), PDS000171574.16 (Israel), PDS000056139.15 (Saudi Arabia), and PDS000166904.10 (Saudi Arabia), were exclusively country-specific. The regionally restricted lineage identified in this study may be due to localized transmission networks, hospital outbreaks, or country-specific selective pressures, such as antimicrobial use [34]. Other factors, including variations in stewardship practices and healthcare infrastructure across countries, may also shape which clones become dominant. Country-specific studies are crucial for identifying the fundamental reasons behind this phenomenon.

On the other hand, a more dispersed distribution was also found for the other clusters. For instance, PDS000012112.206 was detected in four countries of the MENA region, with the majority of isolates belonging to Saudi Arabia and Egypt, followed by Jordan and Oman. The other cluster, PDS000060581.77, was predominantly detected in Saudi Arabia, with low prevalence in Egypt, Oman, and Tunisia. Factors such as rapid urban growth, intensive use of healthcare services, numerous international travels, and climate change may contribute to the regional or global spread of infectious diseases [50,51,52,53,54,55]. Incorporating travel-associated genomic surveillance may be valuable in interpreting regional transmission patterns and explaining the dispersed distribution of lineages across countries.

Interestingly, our findings indicated that Saudi Arabia was the only country in the MENA region where five of the top 10 clusters were detected, consisting of an almost-high prevalence of both country-specific and dispersed clusters. This could be partially explained by the large-scale events, such as the annual Muslim pilgrimage, Hajj, and Umrah in this country, which may contribute to the dissemination of *K. pneumoniae* strains [56]. Genomic surveillance during and after mass gatherings may provide insight into the contribution of these events to the international spread of pathogens. Future investigations are needed to clarify other underlying reasons.

We found a higher distribution of antimicrobial-resistant genes across the top 10 *K. pneumoniae* SNP clusters. This may be concerning, given the detection of multidrug resistance, such as *blaSHV-1*, *blaCTX-M-15*, and *blaNDM-1*, which may contribute to the burden of healthcare-associated infections [9,18,49]. Additionally, the co-occurrence of plasmid-mediated colistin resistance genes in clusters underscores possible limitations of therapeutic options. According to the previous genomic studies, rare lineages can be reservoirs of novel mobile genetic elements and antimicrobial resistance determinants [57] that may later be acquired by dominant clones. Therefore, targeted genomic characterization of all clusters, including singleton and low-frequency clusters, should be investigated to detect emerging threats before they expand.

Our geographical analysis highlighted both localized persistence and broader dissemination of SNP clusters of *K. pneumoniae*. These findings were consistent with previous studies. While some reports describe the rapid spread of particular *K. pneumoniae* strains across regions or countries [58,59,60], others focus on localized outbreaks within single hospitals [48,61,62]. Although the focus of this study was on dominant clusters, rare clusters also need important attention as they may be clinically significant, particularly if they carry novel antimicrobial resistance genes or virulence factors. Integrating antimicrobial use metrics with genomic data may also help determine how prescribing behavior influences the distribution of clones. Further studies are warranted to specifically investigate the spatial distribution of regional rare clusters and carrying resistance genes.

Our MLST analysis indicated the presence of high-risk clones (ST11, ST258, ST512) among *K. pneumoniae* clinical isolates from the MENA region, which was in agreement with what was reported in a recent investigation in Europe [27].

This study is not free of limitations. Since this study used publicly available data from NCBI, the dataset may not represent uniform or systematic sampling across countries or years. Accordingly, the results should be interpreted as descriptive of the available data. This study provided yearly prevalence estimates for the top clusters rather than conducting a temporal trend analysis, due to inconsistent detection across all years of the study period. Moreover, our ability to correlate cluster prevalences with patient outcomes was limited due to the lack of clinical metadata. Finally, the uneven number of isolates across countries may have affected the geographical representation of the top clusters.

## 4. Materials and Methods

### 4.1. Data Collection and Preprocessing

**Figure 5:**
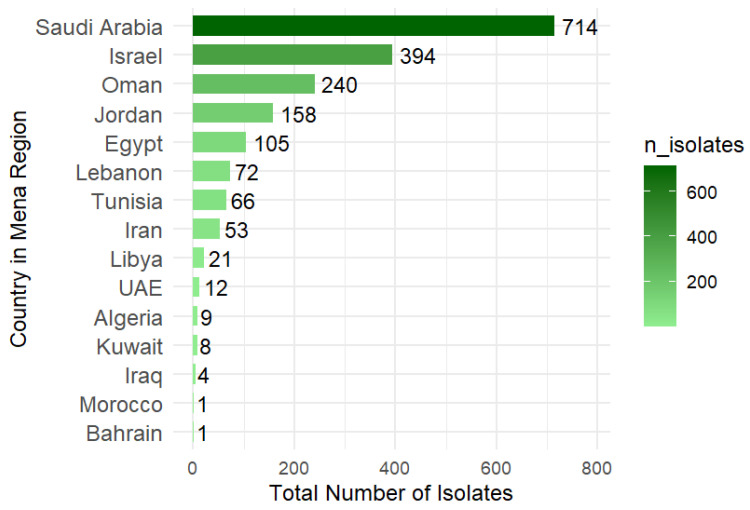
Total number of clinical *K. pneumoniae* isolates from 15 MENA countries obtained from the NCBI database between 2018 and 2024. The number across the bar represents the number of isolates.

In this study, we utilized the NCBI Pathogen Detection database (https://www.ncbi.nlm.nih.gov/pathogens/; accessed on 25 March 2026) to extract data on clinical *K. pneumoniae* isolates recovered from countries in the Middle East and North Africa (MENA) between 8 January 2018 and 11 December 2024 (Table S1). This study excluded the isolates without SNP cluster identifier information from the dataset. A total of 1858 isolates belonging to 15 countries extracted and included in this study are shown in Figure 1. The highest representation was from Saudi Arabia (714 isolates, 38.4%), followed by Israel (394 isolates, 21.2%), Oman (240 isolates, 12.9%), Jordan (158 isolates, 8.5%), and Egypt (105 isolates, 5.7%). Smaller numbers of isolates were obtained from Lebanon, Tunisia, Iran, Libya, UAE, Algeria, Kuwait, Iraq, Bahrain, and Morocco (Figure 5).

In the present study, we performed descriptive and regression analyses and visualized them using R (version 4.5.0) software within the RStudio environment (2024.09.0 Build 375, Posit Software) [63].

### 4.2. Overall Cluster Prevalence

The prevalence of each SNP cluster among the reported *Klebsiella* isolates was calculated as the number of isolates carrying each cluster divided by the total number of isolates. A summary table of overall cluster prevalence was generated and presented as a Supplementary File.

### 4.3. Identification of Top 10 SNP Clusters

The ten most prevalent SNP clusters were identified based on their proportions calculated in Section 4.2. Isolates belonging to these top 10 clusters were subsetted for detailed analysis. The prevalence of each of the top 10 clusters was calculated and visualized as horizontal bar plots using the ggplot2 R package (ver 4.0.0) to identify the dominant clusters.

### 4.4. Descriptive Yearly Snapshots of Top 10 SNP Clusters

The prevalence of the top 10 clusters was calculated for each year of the study period (2018–2024) to provide descriptive snapshots of temporal distribution. The results were then visualized in bar plots for each top cluster using the ggplot2 R package. Yearly prevalence estimates of the top clusters, rather than temporal trend analysis, were used because the detection of the top clusters was inconsistent across all years of the study period.

### 4.5. Geographic Distribution of Top 10 SNP Clusters

For each of the top 10 clusters, the number and proportion of isolates per country were calculated to assess geographic concentration. To visualize the distribution, geographic maps of MENA countries were generated using the sf and rnaturalearth R packages (ver 1.1.0). The top SNP cluster proportions were shown using faceted maps with gradient color fills to illustrate the relative distribution of each cluster across the MENA region.

### 4.6. AMR Gene Prevalence in SNP Clusters

The AMR genotypes in the databases were parsed to identify the presence of key resistance genes associated with carbapenem (*blaKPC*, *blaNDM*, and *blaOXA* variants), ESBL *(blaCTX-M*, *blaSHV*, and *blaTEM* variants), and colistin (*mcr*, *mgr*, *pmrA*, and *pmrB* variants) resistance. Although multiple types of resistance genes have been reported, we have identified only these three clinically and globally resistant genes, which help track the spread of high-risk clones that affect treatment options. For each top 10 SNP cluster, the first isolates within each cluster that carry the respective genes were counted. Then, frequencies were normalized by calculating the percentage of isolates within each cluster carrying each resistance gene. This is visualized using a heatmap and hierarchical clustering dendrogram in R, generated with the pheatmap package (ver 1.0.13).

### 4.7. MLST Across Top 10 SNP Clusters

From the dataset, accession numbers of the representative isolates for each of the top 10 clusters were used to download genome FASTA files from NCBI. MLST analysis was performed in a Linux environment (Ubuntu 24.04) using MLST tool version 2.25.0 to assign sequence types (STs) to these clusters [64].

## 5. Conclusions

The present study provided genomic epidemiology of *K. pneumoniae* in the MENA region from 2018 to 2024. Heterogeneous population structure was identified among the isolates. Among 478 identified SNP clusters, a few dominant clusters accounted for 37% of the isolates, and numerous low-frequency lineages were detected. Our descriptive yearly snapshot revealed a diverse representation of top clusters. Geographical analysis revealed both localized and limited cross-border distribution patterns. We suggest further longitudinal studies on both dominant and rare clusters of *K. pneumoniae* isolates from the MENA region to better characterize temporal and regional changes. To better understand the public health implications of these lineages, it is recommended that genomic data be integrated with clinical and antimicrobial resistance information. Furthermore, expanding sampling across underrepresented countries in the MENA region is also suggested to identify unknown clonal diversity and transmission routes. Finally, studies on rare lineages are also important for identifying their clinical significance, as they may harbor novel resistance genes and virulence factors.

## Figures and Tables

**Table 1:** Geographical distribution of the top 10 SNP clusters and associated MLST of clinical *K. pneumoniae* isolated from MENA countries between 2018 and 2024.

SNP Cluster	Sequence Types *	Location	Number of Isolates	Prevalence (%)
PDS000012112.206	ST219	Saudi Arabia	10	52.63
		Egypt	6	31.58
		Jordan	2	10.53
		Oman	1	5.2
PDS000046911.4	ST11	Oman	61	100
PDS000056139.15	ST14	Saudi Arabia	38	100
PDS000060581.77	ST2096	Saudi Arabia	137	97.86
		Egypt	1	0.71
		Oman	1	0.71
		Tunisia	1	0.71
PDS000105033.2	ST512	Israel	108	100
PDS000161007.19	ST11	Saudi Arabia	15	65.22
		Oman	4	17.39
		Libya	3	13.04
		Egypt	1	4.35
PDS000166904.10	ST2096	Saudi Arabia	32	100
PDS000171574.16	ST258	Israel	160	100
PDS000219448.13	ST231	Oman	78	100
PDS000240474.1	ST147	Oman	16	55.17
		Jordan	10	34.48
		Egypt	3	10.34

* Multi-locus sequence types were determined for each SNP cluster, using FASTA files via MLSTtools.

## Data Availability

Data Availability Statement indicates that the data supporting our findings are publicly available from the NCBI Pathogen Isolates database, with the relevant URL provided.

## Supplementary Materials

The following supporting information can be downloaded at: https://www.mdpi.com/article/10.3390/antibiotics15040349/s1.
