# Supplementary material for: Genomic Epidemiology of Clinical Klebsiella pneumoniae in the Middle East and North Africa
Source: Antibiotics (Basel). 2026 Mar 29;15(4):349. doi: 10.3390/antibiotics15040349 (PMC13113317; doi:10.3390/antibiotics15040349)
Supplement: Supplementary file 1 [file antibiotics-15-00349-s001.zip › Tables 2 and 3.pdf]

Table S2. Prevalence of SNP clusters of *K. pneumoniae* in the MENA region (2018–2024).

SNP cluster	Number of_isolates	Prevalence
PDS000171574.16	160	0.086114
PDS000060581.77	140	0.07535
PDS000105033.2	108	0.058127
PDS000219448.13	78	0.041981
PDS000046911.4	61	0.032831
PDS000056139.15	38	0.020452
PDS000166904.10	32	0.017223
PDS000240474.1	29	0.015608
PDS000161007.19	23	0.012379
PDS000012112.206	19	0.010226
PDS000006578.133	18	0.009688
PDS000046919.12	18	0.009688
PDS000185299.8	17	0.00915
PDS000188947.32	17	0.00915
PDS000117506.31	14	0.007535
PDS000231673.7	14	0.007535
PDS000104512.15	13	0.006997
PDS000164238.2	13	0.006997
PDS000141863.2	12	0.006459
PDS000100154.33	11	0.00592
PDS000045131.5	10	0.005382
PDS000085592.12	10	0.005382
PDS000201306.1	10	0.005382
PDS000045427.1	9	0.004844
PDS000100344.18	9	0.004844
PDS000149421.5	9	0.004844
PDS000156348.4	9	0.004844
PDS000046910.95	8	0.004306
PDS000060651.41	8	0.004306
PDS000061038.3	8	0.004306
PDS000104567.35	8	0.004306
PDS000108802.3	8	0.004306
PDS000109683.33	8	0.004306
PDS000045135.27	7	0.003767
PDS000046311.33	7	0.003767
PDS000060601.1	7	0.003767
PDS000060610.1	7	0.003767
PDS000075319.9	7	0.003767

PDS000080100.1	7	0.003767
PDS000112591.1	7	0.003767
PDS000166912.7	7	0.003767
PDS000171214.1	7	0.003767
PDS000176486.3	7	0.003767
PDS000041740.153	6	0.003229
PDS000060649.2	6	0.003229
PDS000079862.22	6	0.003229
PDS000084235.3	6	0.003229
PDS000090167.16	6	0.003229
PDS000091501.235	6	0.003229
PDS000100093.143	6	0.003229
PDS000189621.29	6	0.003229
PDS000219437.3	6	0.003229
PDS000012132.13	5	0.002691
PDS000060597.1	5	0.002691
PDS000060609.1	5	0.002691
PDS000060615.1	5	0.002691
PDS000076063.73	5	0.002691
PDS000093787.1	5	0.002691
PDS000112585.1	5	0.002691
PDS000122484.31	5	0.002691
PDS000166495.8	5	0.002691
PDS000166905.6	5	0.002691
PDS000176485.1	5	0.002691
PDS000176497.1	5	0.002691
PDS000176521.7	5	0.002691
PDS000176524.1	5	0.002691
PDS000041735.8	4	0.002153
PDS000044310.7	4	0.002153
PDS000045417.4	4	0.002153
PDS000046916.2	4	0.002153
PDS000046917.1	4	0.002153
PDS000055637.4	4	0.002153
PDS000058605.3	4	0.002153
PDS000060607.1	4	0.002153
PDS000065989.3	4	0.002153
PDS000075316.3	4	0.002153
PDS000077095.1	4	0.002153
PDS000084053.3	4	0.002153
PDS000084610.2	4	0.002153
PDS000101822.6	4	0.002153
PDS000106322.3	4	0.002153

PDS000112586.1	4	0.002153
PDS000112587.1	4	0.002153
PDS000164521.1	4	0.002153
PDS000166494.11	4	0.002153
PDS000176484.3	4	0.002153
PDS000176495.1	4	0.002153
PDS000176500.3	4	0.002153
PDS000185321.4	4	0.002153
PDS000192496.4	4	0.002153
PDS000193784.2	4	0.002153
PDS000199419.2	4	0.002153
PDS000199425.2	4	0.002153
PDS000199426.1	4	0.002153
PDS000201443.3	4	0.002153
PDS000042807.4	3	0.001615
PDS000045324.14	3	0.001615
PDS000052090.1	3	0.001615
PDS000052095.1	3	0.001615
PDS000060412.6	3	0.001615
PDS000060594.2	3	0.001615
PDS000060749.7	3	0.001615
PDS000065988.2	3	0.001615
PDS000075296.17	3	0.001615
PDS000085673.3	3	0.001615
PDS000100551.2	3	0.001615
PDS000102373.5	3	0.001615
PDS000104062.40	3	0.001615
PDS000106324.3	3	0.001615
PDS000145286.2	3	0.001615
PDS000161014.2	3	0.001615
PDS000164530.1	3	0.001615
PDS000164531.1	3	0.001615
PDS000173135.2	3	0.001615
PDS000176503.3	3	0.001615
PDS000176507.1	3	0.001615
PDS000176514.3	3	0.001615
PDS000185300.1	3	0.001615
PDS000185303.4	3	0.001615
PDS000185304.1	3	0.001615
PDS000185306.3	3	0.001615
PDS000185309.1	3	0.001615
PDS000185310.6	3	0.001615
PDS000185317.2	3	0.001615

PDS000186869.3	3	0.001615
PDS000192486.2	3	0.001615
PDS000201444.2	3	0.001615
PDS000201449.3	3	0.001615
PDS000205503.4	3	0.001615
PDS000205536.1	3	0.001615
PDS000205544.1	3	0.001615
PDS000225936.2	3	0.001615
PDS000235542.1	3	0.001615
PDS000005975.20	2	0.001076
PDS000018116.1	2	0.001076
PDS000018122.1	2	0.001076
PDS000041726.21	2	0.001076
PDS000045134.1	2	0.001076
PDS000045260.46	2	0.001076
PDS000045329.20	2	0.001076
PDS000045344.11	2	0.001076
PDS000045571.1	2	0.001076
PDS000045572.1	2	0.001076
PDS000051033.1	2	0.001076
PDS000052091.1	2	0.001076
PDS000052093.1	2	0.001076
PDS000052094.1	2	0.001076
PDS000055645.1	2	0.001076
PDS000060593.1	2	0.001076
PDS000060595.1	2	0.001076
PDS000060596.1	2	0.001076
PDS000060598.1	2	0.001076
PDS000060599.1	2	0.001076
PDS000060602.1	2	0.001076
PDS000060603.1	2	0.001076
PDS000060605.1	2	0.001076
PDS000060606.1	2	0.001076
PDS000060608.3	2	0.001076
PDS000060611.1	2	0.001076
PDS000060612.1	2	0.001076
PDS000060613.1	2	0.001076
PDS000060616.3	2	0.001076
PDS000073393.5	2	0.001076
PDS000074843.3	2	0.001076
PDS000075509.1	2	0.001076
PDS000075528.2	2	0.001076
PDS000079301.1	2	0.001076

PDS000079303.1	2	0.001076
PDS000079305.1	2	0.001076
PDS000080305.7	2	0.001076
PDS000085674.1	2	0.001076
PDS000092819.12	2	0.001076
PDS000093260.14	2	0.001076
PDS000093781.1	2	0.001076
PDS000095134.1	2	0.001076
PDS000097168.2	2	0.001076
PDS000100374.16	2	0.001076
PDS000108801.2	2	0.001076
PDS000111777.1	2	0.001076
PDS000111778.2	2	0.001076
PDS000112584.1	2	0.001076
PDS000112588.1	2	0.001076
PDS000112589.1	2	0.001076
PDS000112590.1	2	0.001076
PDS000114900.3	2	0.001076
PDS000117819.1	2	0.001076
PDS000145287.1	2	0.001076
PDS000145289.1	2	0.001076
PDS000155792.1	2	0.001076
PDS000157722.1	2	0.001076
PDS000161008.1	2	0.001076
PDS000161009.1	2	0.001076
PDS000161015.1	2	0.001076
PDS000161018.1	2	0.001076
PDS000164246.1	2	0.001076
PDS000164518.1	2	0.001076
PDS000164519.1	2	0.001076
PDS000166925.4	2	0.001076
PDS000166926.2	2	0.001076
PDS000169474.24	2	0.001076
PDS000171211.1	2	0.001076
PDS000173803.4	2	0.001076
PDS000176483.1	2	0.001076
PDS000176489.1	2	0.001076
PDS000176493.1	2	0.001076
PDS000176494.1	2	0.001076
PDS000176496.1	2	0.001076
PDS000176499.1	2	0.001076
PDS000176502.1	2	0.001076
PDS000176505.1	2	0.001076

PDS000176513.1	2	0.001076
PDS000176515.1	2	0.001076
PDS000176519.1	2	0.001076
PDS000176520.1	2	0.001076
PDS000178881.6	2	0.001076
PDS000180182.1	2	0.001076
PDS000180183.1	2	0.001076
PDS000180184.1	2	0.001076
PDS000185297.1	2	0.001076
PDS000185305.2	2	0.001076
PDS000185307.1	2	0.001076
PDS000185308.2	2	0.001076
PDS000185314.1	2	0.001076
PDS000185315.3	2	0.001076
PDS000185316.1	2	0.001076
PDS000185320.1	2	0.001076
PDS000185322.2	2	0.001076
PDS000186873.1	2	0.001076
PDS000188993.3	2	0.001076
PDS000193783.2	2	0.001076
PDS000199420.1	2	0.001076
PDS000199421.1	2	0.001076
PDS000199428.1	2	0.001076
PDS000199430.1	2	0.001076
PDS000201447.1	2	0.001076
PDS000201452.1	2	0.001076
PDS000201454.1	2	0.001076
PDS000201456.2	2	0.001076
PDS000201460.2	2	0.001076
PDS000201464.1	2	0.001076
PDS000205497.2	2	0.001076
PDS000205500.2	2	0.001076
PDS000205504.2	2	0.001076
PDS000205508.1	2	0.001076
PDS000205510.2	2	0.001076
PDS000205513.2	2	0.001076
PDS000205515.1	2	0.001076
PDS000205520.3	2	0.001076
PDS000205521.1	2	0.001076
PDS000205522.2	2	0.001076
PDS000205524.1	2	0.001076
PDS000205526.1	2	0.001076
PDS000205527.2	2	0.001076

PDS000205528.3	2	0.001076
PDS000205530.1	2	0.001076
PDS000205532.1	2	0.001076
PDS000205533.1	2	0.001076
PDS000205537.3	2	0.001076
PDS000205541.1	2	0.001076
PDS000205542.1	2	0.001076
PDS000205545.2	2	0.001076
PDS000225804.1	2	0.001076
PDS000225829.1	2	0.001076
PDS000225832.2	2	0.001076
PDS000225848.1	2	0.001076
PDS000225852.1	2	0.001076
PDS000225854.1	2	0.001076
PDS000225855.1	2	0.001076
PDS000225905.1	2	0.001076
PDS000225909.2	2	0.001076
PDS000225963.2	2	0.001076
PDS000225981.1	2	0.001076
PDS000234238.1	2	0.001076
PDS000005615.31	1	0.000538
PDS000013916.2	1	0.000538
PDS000017392.17	1	0.000538
PDS000019838.1	1	0.000538
PDS000036288.4	1	0.000538
PDS000038919.6	1	0.000538
PDS000039681.1	1	0.000538
PDS000040457.37	1	0.000538
PDS000040464.11	1	0.000538
PDS000045274.52	1	0.000538
PDS000045314.7	1	0.000538
PDS000045384.36	1	0.000538
PDS000045409.8	1	0.000538
PDS000045430.15	1	0.000538
PDS000052092.1	1	0.000538
PDS000053905.24	1	0.000538
PDS000056119.1	1	0.000538
PDS000056167.11	1	0.000538
PDS000059962.29	1	0.000538
PDS000060414.77	1	0.000538
PDS000060614.1	1	0.000538
PDS000060654.31	1	0.000538
PDS000070252.8	1	0.000538

PDS000074892.1	1	0.000538
PDS000075204.13	1	0.000538
PDS000075208.6	1	0.000538
PDS000075507.3	1	0.000538
PDS000075508.6	1	0.000538
PDS000076562.102	1	0.000538
PDS000077168.1	1	0.000538
PDS000077829.4	1	0.000538
PDS000079304.11	1	0.000538
PDS000080297.2	1	0.000538
PDS000085652.2	1	0.000538
PDS000090379.3	1	0.000538
PDS000093783.1	1	0.000538
PDS000097160.5	1	0.000538
PDS000098809.6	1	0.000538
PDS000100125.8	1	0.000538
PDS000101810.3	1	0.000538
PDS000101844.1	1	0.000538
PDS000101906.4	1	0.000538
PDS000102341.9	1	0.000538
PDS000103198.2	1	0.000538
PDS000104546.2	1	0.000538
PDS000106388.2	1	0.000538
PDS000106697.8	1	0.000538
PDS000108016.1	1	0.000538
PDS000108275.13	1	0.000538
PDS000108393.5	1	0.000538
PDS000110900.1	1	0.000538
PDS000114177.1	1	0.000538
PDS000114328.3	1	0.000538
PDS000124937.1	1	0.000538
PDS000124949.4	1	0.000538
PDS000129327.49	1	0.000538
PDS000130861.2	1	0.000538
PDS000143133.1	1	0.000538
PDS000156641.21	1	0.000538
PDS000158001.18	1	0.000538
PDS000160828.3	1	0.000538
PDS000160830.3	1	0.000538
PDS000161016.1	1	0.000538
PDS000164532.2	1	0.000538
PDS000166486.7	1	0.000538
PDS000166487.1	1	0.000538

PDS000166492.8	1	0.000538
PDS000166916.7	1	0.000538
PDS000166917.2	1	0.000538
PDS000170608.1	1	0.000538
PDS000171212.1	1	0.000538
PDS000173787.2	1	0.000538
PDS000175702.4	1	0.000538
PDS000175846.1	1	0.000538
PDS000176509.1	1	0.000538
PDS000178883.1	1	0.000538
PDS000179249.1	1	0.000538
PDS000185318.1	1	0.000538
PDS000185326.3	1	0.000538
PDS000186877.2	1	0.000538
PDS000192487.4	1	0.000538
PDS000192610.1	1	0.000538
PDS000193785.1	1	0.000538
PDS000199415.3	1	0.000538
PDS000199417.1	1	0.000538
PDS000199423.1	1	0.000538
PDS000199427.2	1	0.000538
PDS000200955.1	1	0.000538
PDS000201448.1	1	0.000538
PDS000202473.2	1	0.000538
PDS000205499.1	1	0.000538
PDS000205525.2	1	0.000538
PDS000205539.1	1	0.000538
PDS000205547.1	1	0.000538
PDS000206361.1	1	0.000538
PDS000209760.3	1	0.000538
PDS000212920.1	1	0.000538
PDS000213208.2	1	0.000538
PDS000214277.2	1	0.000538
PDS000214320.5	1	0.000538
PDS000214762.2	1	0.000538
PDS000225865.2	1	0.000538
PDS000225871.1	1	0.000538
PDS000225914.2	1	0.000538
PDS000228396.2	1	0.000538
PDS000228397.2	1	0.000538
PDS000228407.1	1	0.000538
PDS000228433.1	1	0.000538
PDS000230722.1	1	0.000538

PDS000231252.2	1	0.000538
PDS000231490.1	1	0.000538
PDS000231492.1	1	0.000538
PDS000231493.1	1	0.000538
PDS000231495.1	1	0.000538
PDS000231496.1	1	0.000538
PDS000231501.1	1	0.000538
PDS000231506.1	1	0.000538
PDS000231507.2	1	0.000538
PDS000231510.1	1	0.000538
PDS000234188.1	1	0.000538
PDS000234189.1	1	0.000538
PDS000234190.1	1	0.000538
PDS000234192.1	1	0.000538
PDS000234193.1	1	0.000538
PDS000234194.1	1	0.000538
PDS000234195.1	1	0.000538
PDS000234196.1	1	0.000538
PDS000234197.1	1	0.000538
PDS000234198.1	1	0.000538
PDS000234199.2	1	0.000538
PDS000234200.1	1	0.000538
PDS000234201.1	1	0.000538
PDS000234203.1	1	0.000538
PDS000234204.1	1	0.000538
PDS000234207.1	1	0.000538
PDS000234208.1	1	0.000538
PDS000234209.1	1	0.000538
PDS000234211.1	1	0.000538
PDS000234212.1	1	0.000538
PDS000234213.1	1	0.000538
PDS000234214.1	1	0.000538
PDS000234216.1	1	0.000538
PDS000234217.1	1	0.000538
PDS000234218.1	1	0.000538
PDS000234219.1	1	0.000538
PDS000234220.1	1	0.000538
PDS000234222.1	1	0.000538
PDS000234223.1	1	0.000538
PDS000234224.1	1	0.000538
PDS000234225.1	1	0.000538
PDS000234226.1	1	0.000538
PDS000234227.1	1	0.000538

PDS000234228.1	1	0.000538
PDS000234229.1	1	0.000538
PDS000234230.1	1	0.000538
PDS000234231.1	1	0.000538
PDS000234234.1	1	0.000538
PDS000234235.1	1	0.000538
PDS000234236.1	1	0.000538
PDS000234240.1	1	0.000538
PDS000234241.1	1	0.000538
PDS000234242.1	1	0.000538
PDS000234243.1	1	0.000538
PDS000234244.1	1	0.000538
PDS000234247.1	1	0.000538
PDS000234248.1	1	0.000538
PDS000234249.1	1	0.000538
PDS000234250.1	1	0.000538
PDS000234251.1	1	0.000538
PDS000234252.1	1	0.000538
PDS000234253.1	1	0.000538
PDS000234254.1	1	0.000538
PDS000234255.1	1	0.000538
PDS000234256.1	1	0.000538
PDS000234258.1	1	0.000538
PDS000234259.1	1	0.000538
PDS000234260.2	1	0.000538
PDS000234263.1	1	0.000538
PDS000234264.1	1	0.000538
PDS000234266.1	1	0.000538
PDS000234267.1	1	0.000538
PDS000234268.1	1	0.000538
PDS000234269.1	1	0.000538
PDS000234271.1	1	0.000538
PDS000234272.1	1	0.000538
PDS000234273.1	1	0.000538
PDS000234274.1	1	0.000538
PDS000234275.1	1	0.000538
PDS000234276.1	1	0.000538
PDS000234278.1	1	0.000538
PDS000234279.1	1	0.000538
PDS000234280.1	1	0.000538
PDS000234281.1	1	0.000538
PDS000234282.1	1	0.000538
PDS000234284.1	1	0.000538

PDS000234286.1	1	0.000538
PDS000234287.1	1	0.000538
PDS000234288.1	1	0.000538
PDS000234289.1	1	0.000538
PDS000234290.1	1	0.000538
PDS000234291.1	1	0.000538
PDS000234295.1	1	0.000538
PDS000234807.1	1	0.000538
PDS000235553.1	1	0.000538
PDS000239497.1	1	0.000538

Table S3. Prevalence of the top 10 clusters of clinical *K. pneumoniae* isolated from MENA countries between 2018 and 2024.

Year	SNP cluster	Number of Isolates	Prevalence
2018	PDS000105033.2	99	0.386719
2018	PDS000171574.16	157	0.613281
2019	PDS000012112.206	1	0.006329
2019	PDS000046911.4	61	0.386076
2019	PDS000060581.77	1	0.006329
2019	PDS000105033.2	4	0.025316
2019	PDS000171574.16	2	0.012658
2019	PDS000219448.13	71	0.449367
2019	PDS000240474.1	18	0.113924
2020	PDS000056139.15	31	0.256198
2020	PDS000060581.77	86	0.710744
2020	PDS000105033.2	4	0.033058
2021	PDS000056139.15	3	0.3
2021	PDS000060581.77	1	0.1
2021	PDS000161007.19	3	0.3
2021	PDS000171574.16	1	0.1
2021	PDS000219448.13	1	0.1
2021	PDS000240474.1	1	0.1
2022	PDS000060581.77	3	0.75
2022	PDS000105033.2	1	0.25
2023	PDS000060581.77	4	0.125
2023	PDS000161007.19	11	0.34375
2023	PDS000166904.10	11	0.34375
2023	PDS000219448.13	6	0.1875
2024	PDS000012112.206	18	0.168224
2024	PDS000056139.15	4	0.037383
2024	PDS000060581.77	45	0.420561
2024	PDS000161007.19	9	0.084112
2024	PDS000166904.10	21	0.196262
2024	PDS000240474.1	10	0.093458
